# Engineering of Pyranose Dehydrogenase for Increased Oxygen Reactivity

**DOI:** 10.1371/journal.pone.0091145

**Published:** 2014-03-10

**Authors:** Iris Krondorfer, Katharina Lipp, Dagmar Brugger, Petra Staudigl, Christoph Sygmund, Dietmar Haltrich, Clemens K. Peterbauer

**Affiliations:** 1 Food Biotechnology Laboratory, Department of Food Science and Technology, University of Natural Resources and Life Sciences, Vienna, Austria; 2 University of Applied Sciences Wiener Neustadt – Campus Tulln, Tulln, Austria; Jacobs University Bremen, Germany

## Abstract

Pyranose dehydrogenase (PDH), a member of the GMC family of flavoproteins, shows a very broad sugar substrate specificity but is limited to a narrow range of electron acceptors and reacts extremely slowly with dioxygen as acceptor. The use of substituted quinones or (organo)metals as electron acceptors is undesirable for many production processes, especially of food ingredients. To improve the oxygen reactivity, site-saturation mutagenesis libraries of twelve amino acids around the active site of *Agaricus meleagris* PDH were expressed in *Saccharomyces cerevisiae*. We established high-throughput screening assays for oxygen reactivity and standard dehydrogenase activity using an indirect Amplex Red/horseradish peroxidase and a DCIP/D-glucose based approach. The low number of active clones confirmed the catalytic role of H512 and H556. Only one position was found to display increased oxygen reactivity. Histidine 103, carrying the covalently linked FAD cofactor in the wild-type, was substituted by tyrosine, phenylalanine, tryptophan and methionine. Variant H103Y was produced in *Pichia pastoris* and characterized and revealed a five-fold increase of the oxygen reactivity.

## Introduction

The flavoenzyme pyranose dehydrogenase (PDH, EC 1.1.99.29), a monomeric, extracellular fungal glycoprotein of around 65 kDa, catalyzes the oxidation of a broad range of mono-, oligosaccharides and glycosides at different sites to the corresponding aldoketose or diketose derivatives. Dependent on the sugar, the source of the enzyme and the reaction conditions, C-1, C-2, C-3 and dioxidations at C-1,2, C-2,3 and C-3,4 can be performed. Unlike the closely related enzyme pyranose 2-oxidase (POx), PDH is unable to utilize O_2_ as an electron acceptor, (substituted) quinones and complexed metal ions are used instead [1].

PDH was first isolated from the edible basidiomycete *Agaricus bisporus* and subsequently from other members of the *Agaricaceae* family [2]–[6]. The fungus *A. meleagris* was found to possess three *pdh*-genes, with *pdh1* being transcribed in higher levels than *pdh2* and *pdh3*, especially in stages of oxygen deprivation [7]. POx- and PDH-encoding genes were found to occur mutually exclusive in Basidiomycetes and have not been detected in one species. Wood-degrading white rot fungi (e.g. *Trametes sp.*, *Phanerochaete sp*.) generally possess POx whereas PDH seems to be limited to the mostly litter-decomposing fungi of the family *Agaricaceae* [3]. The biological function of the enzyme is not clear, due to the narrow range of electron acceptors one role of PDH could be the reduction of reactive radicals and quinone compounds emerging during lignin decomposition to prevent their repolymerization and protect the cell from damage [8]–[10].

Its substrate promiscuity renders the enzyme a promising biocatalyst for the synthesis of high-value carbohydrate compounds in the pharmaceutical-, cosmetic- and food industry [5], [6], [11], [12]. Removal of the (mostly) undesirable electron acceptors would increase costs of product purification significantly and limit the application of PDH in industrial scale, especially for the production of food ingredients. Despite the disadvantageous effects of the produced hydrogen peroxide, an enzyme with the broad sugar substrate specificity of PDH utilizing O_2_ as a nonhazardous electron acceptor could substantially simplify production processes.

The overall structure of PDH shows a typical p-hydroxybenzoate hydroxylase (PHBH)-like fold that is shared by the members of the glucose-methanol-choline (GMC) family of oxidoreductases like POx, glucose oxidase (GOx) or cellobiose dehydrogenase (CDH). PDH consists of a Rossman-domain where the FAD cofactor is covalently bound and a substrate binding domain [13].

Several structural characteristics in flavoprotein oxidases apparently contribute to oxygen reactivity, but a general mechanism why a protein does or does not react with oxygen remains unknown [14]. The chemistry of the reaction with oxygen is believed to start with an initial electron transfer from the reduced flavin to oxygen, which forms O_2_^-•^ and flavin semiquinone. In flavoprotein oxidases an immediate second electron transfer occurs, resulting in the reoxidized flavin and H_2_O_2_. Monooxygenases and certain oxidases like POx form a transient C(4a)-(hydro)peroxyflavin intermediate [15], [16]. In the recently resolved crystal structure of PDH, such a C(4a) adduct is visible as well. Although considered a radiation artifact, it shows that PDH is able to form and stabilize such an adduct [13].

A protonated histidine in the active site of GOx was identified to be crucial for the first and rate-limiting electron transfer step [17], [18]. Studies on other flavoprotein oxidases showed a positive charge in the active site to be a general prerequisite [19]. Not only amino acids, like a lysine in monomeric sarcosine oxidase [20], but also a charged substrate like in choline oxidase can fulfill this requirement [21]. Furthermore, a nonpolar site close to the flavin C(4a) in addition to the positive charge seems to maximize these electrostatic effects by desolvation of the active site [19], [22]. The accessibility of the active site through tunnels and channels is another important aspect for oxygen reactivity [23]–[25]. All these observations indicate that the microenvironment of the flavin, especially of the C(4a)N5 locus, and subtle changes therein, are of major importance concerning oxygen reactivity [26].

For this reason a semi-rational approach using site-saturation mutagenesis of twelve amino acids in the active site of *A. meleagris* PDH (AmPDH) was chosen for this work. We show for the first time the expression of site-saturation libraries of AmPDH in *S. cerevisiae* and the high-throughput screening for increased oxygen reactivity using an Amplex Red/horseradish peroxidase based assay. Amino acid substitutions only at position H103 to tyrosine, phenylalanine, tryptophan and methionine showed increased oxygen reactivity. Variant H103Y was produced in 5 L scale and partially characterized in order to gain further insight into the oxygen reactivity of flavoproteins.

## Material and Methods

### 2.1 Chemicals and vectors

All chemicals were of the highest purity available and purchased from Sigma Aldrich (St. Louis, MO), Roth (Karlsruhe, Germany), and VWR (Radnor, PA). Primers were from LGC genomics (Vienna, Austria), restriction endonucleases, T4 DNA ligase and phusion polymerase from Thermo Fisher Scientific Biosciences (St. Leon-Rot, Germany). GoTaq polymerase was obtained from Promega (Madison, WI). The uracil independent and ampicillin resistance-encoding shuttle vector pYES2/CT and the Zeocin resistance-encoding shuttle vector pPICZB were purchased from Invitrogen (Carlsbad, CA).

### 2.2 Strains and media

*P. pastoris* strain X33 was purchased from Invitrogen, the protease-deficient *S. cerevisiae* strain BJ5465 from LGC Promochem (Barcelona, Spain) and *Escherichia coli* strain NEB5α from New England Biolabs (Ipswich, MA). YPD plates contained 10 g L^−1^ peptone, 20 g L^−1^ yeast extract, 10 g L^−1^ D-glucose, 20 g L^−1^ agar and 100 mg L^−1^ Zeocin. LB low salt medium contained 10 g L^−1^ peptone from casein, 5 g L^−1^ yeast extract, 5 g L^−1^ NaCl and 25 mg L^−1^ Zeocin. Basal salts medium (Invitrogen) was used for fermentation, containing 0.93 g L^−1^ calcium sulfate, 18.2 g L^−1^ potassium sulfate, 14.9 g L^−1^ magnesium sulfate heptahydrate, 4.13 g L^−1^ potassium hydroxide, 40 g L^−1^ glycerol, 26.7 mL 85% phosphoric acid and 4.35 mL PTM_1_ trace salts per liter. SC-dropout plates contained 1.92 g L^−1^ Y1501 (synthetic dropout medium without uracil), 20 g L^−1^ agar, 100 mL L^−1^ 20% D-glucose, 100 mL L^−1^ 10× YNB and 1 mL L^−1^ chloramphenicol. For the preparation of the liquid medium (without agar), D-glucose was replaced by raffinose. SG/R-CAA expression medium [27] contained 5 g L^−1^ casein hydrolysate, 9.67 g L^−1^ NaH_2_PO_4_.2H_2_O, 6.77 g L^−1^ Na_2_HPO_4_.2H_2_O, 50 mL L^−1^ 10× YNB, 100 mL L^−1^ 20% D-galactose, 100 mL L^−1^ 20% raffinose and 5 mL L^−1^ 20% D-glucose.

### 2.3 Construction of plasmids for expression in P. pastoris and S. cerevisiae

The internal *Bam*HI restriction site of the *Agaricus meleagris pdh1* gene [7] in the vector pCR Blunt II TOPO (Invitrogen) *pdh1* was removed by site-directed mutagenesis following the *Dpn*I-method [28] using the overlapping mutagenesis primers AmFw/AmRev (Table S1). The PCR-product was purified from an agarose gel, digested with *Dpn*I for 2 h at 37°C to degrade methylated template-DNA, and 5 µL of the PCR product were transformed into chemically competent *E. coli* NEB5α. The presence of the mutation was confirmed by sequencing. The *pdh1* gene was then amplified using the primer pairs Am*Kpn*Ifw/Am*Xba*Irev and Am*Bam*HIfw/Am*Not*Irev. The purified product was digested with the respective restriction endonucleases and ligated into the equally treated vectors pPICZB and pYES2/CT, respectively. The resulting plasmids pPICZB_AmPDH and pYES2/CT_AmPDH were transformed into chemically competent *E. coli* NEB5α (New England Biolabs), proliferated and stored at −20°C.

### 2.4 Heterologous expression of A. meleagris PDH in S. cerevisiae

Plasmid pYES2/CT_AmPDH was transformed into *S. cerevisiae* BJ5465 using the yeast transformation kit (Sigma) based on the lithium acetate/ss-DNA/PEG method [29]. Transformed cells were plated onto SC-dropout plates and incubated at 30°C for 4 days. Transformants were picked into 96-well deep-well plates (Ritter, Schwabmünchen, Germany) containing 200 µL of SC-dropout minimal medium. As a negative control, transformants carrying the empty vector pYES2/CT were used (four wells per plate in row 8). The plates were sealed with BreatheEasy membranes (Diversified Biotech, Dedham, MA) to avoid evaporation of the liquid. After 48 h incubation at 25°C and 365 rpm, 500 µL of SG/R-CAA expression medium were added to each well, followed by seven days of incubation under the same conditions.

### 2.5 Site saturation library construction

The plasmid pYES2/CT_AmPDH was used as a template for site-saturation library generation using the overlap extension method [30]. The codon NNK (N = A, G, C, or T and K = G or T) was used to reduce the necessary library size to statistically cover 99% of all possible substitutions [31]. A complementary pair of inner primers was designed for each of the twelve selected positions (Table S1) as well as a pair of outer primers producing overlaps with the vector (RMLN-sense, RMLC-antisense [32]). Two separate PCR reactions were carried out, producing fragment 1 (RMLN-sense and mutagenic reverse-primer) and fragment 2 (mutagenic forward-primer and RMLC-antisense). The two fragments were fused in a third PCR-reaction with the outer (RMLN-sense and RMLC-antisense) primers and the purified PCR-product was transformed to *S. cerevisiae* BJ5465 together with the digested vector pYES2/CT (*Bam*HI, *Not*I) for in-vivo recombination in a 4∶1 ratio. The variant libraries were expressed as described above (section 2.4) for the wildtype enzyme with some modifications: As a negative control two wells in row 8 were inoculated with *S. cerevisiae* transformed with the empty vector pYES2/CT, three wells with *S. cerevisiae* transformants expressing a cellobiose dehydrogenase (CDH) gene from *Myriococcum thermophilum* [33] as a positive control for the Amplex Red oxygen screening assay and three wells with transformants expressing wild-type AmPDH.

### 2.6 High throughput screening assay

One hundred µL of the culture were transferred to a sterile 96-well plate (Greiner, Kremsmünster, Austria) and OD600 was measured. After addition of 100 µL of 30% (v/v) glycerol the plates were stored at −70°C as master plates. The remaining cultures in the deep-well plates were centrifuged for 20 min at 3,000 rpm and room temperature and the screening assay was performed with the supernatant. For each target position 352 clones were screened.

PDH dehydrogenase activity was determined with an assay containing 2,6-dichloroindophenol (DCIP, ε_520_ = 6.8 mM^−1^ cm^−1^) and D-glucose. Fifty µL of the culture supernatant were transferred from the deep-well plate to the 96-well screening plates using the JANUS liquid handling workstation (PerkinElmer, Waltham, MA). The reaction was started by addition of 150 µL of the DCIP assay mixture (300 µM DCIP in 100 mM sodium acetate buffer pH 4 containing 50 mM D-glucose) and the time-dependant reduction of DCIP was followed at 520 nm with an EnSpire plate reader (PerkinElmer). End-point measurements were carried out after 2 h and 4 h incubation at 30°C and the difference in absorbance (ΔAbs) was calculated in relation to wild-type AmPDH. For calculation of the number of active clones all clones with ΔAbs values higher than the negative controls for each screening plate were considered active.

Oxygen reactivity was determined using a fluorimetric assay based on detection of the hydrogen peroxide formed by PDH. *N*-acetyl-3,7-dihydroxyphenoxazine (Amplex Red reagent) and horseradish peroxidase react with hydrogen peroxide and produce highly fluorescent resorufin [34], [35] which was measured at 550 nm excitation and 587 nm emission wavelength. The assay in 50 mM sodium phosphate buffer pH 7.4 contained 25 µM Amplex Red reagent, 10 mM D-glucose, 0.1 U mL^−1^ horseradish peroxidase and 100 µL of culture supernatant. The screening plates were incubated at 30°C for 5 h only in the presence of D-glucose, end-point measurements of the fluorescence were carried out immediately after addition of the Amplex Red reagent.

### 2.7 Rescreening

Aliquots of 5 µL of the best variants from the master plates were used to inoculate 96-well deep-well plates containing 200 µL SC-dropout minimal medium. Five wells were inoculated per clone, in row 6 positive and negative controls were included and the plates were cultivated and screened as described in section 2.5. The clones showing improved oxygen reactivity again were identified by colony-PCR using *Taq* polymerase and the primers RMLN-sense and RMLC-antisense and sequencing of the PCR-product (LGC genomics).

### 2.8 Heterologous expression of H103Y variant in P. pastoris

The mutation H103Y was introduced to the plasmid pPICZB_AmPDH by site-directed mutagenesis as described in section 2.3 using the overlapping primers H103Yfw and H103Yrev. Prior to transformation into electro-competent *P. pastoris* strain X33 the plasmid was linearized with *Pme*I at 37°C for at least 2 h and purified. Transformants were selected on YPD-Zeocin plates and the integration of the gene was verified by colony PCR. PDH variant H103Y was produced in a 7-L fermenter (MBR, Wetzikon, Switzerland) with an initial volume of 4 L basal salts fermentation medium and purified in a 3-step protocol as described before [6]. Fractions of the highest purity were pooled, concentrated and frozen in liquid nitrogen for storage at -30°C. Gel filtration pool 1 was used for all characterizations. Wild-type AmPDH protein was produced and purified as described previously [36]. In contrast to mutant H103Y, AmPDH is present in the reduced state after purification, oxidized enzyme was generated according to [13] except using a Superose 12 column (GE Healthcare, Chalfont St. Giles, UK) and 50 mM potassium phosphate buffer pH 7.5 containing 150 mM NaCl.

### 2.9 Enzyme assay, molecular properties

PDH activity was measured using the standard assay with ferrocenium hexafluorophosphate as described before [6]. Protein concentrations were determined using the method of Bradford with a pre-fabricated assay (BioRad, Hercules, CA), SDS-PAGE and enzymatic deglycosylation using PNGase F were carried out as described previously [36].

Protein concentrations of the samples in the oxidized state were adjusted to an absorbance at 450 nm of around 0.2 with 65 mM sodium phosphate buffer pH 7.5 and an initial spectrum from 300 to 600 nm was recorded using a U-3000 spectrophotometer (Hitachi, Tokyo, Japan). Precipitation was carried out by mixing the double-concentrated protein solution with 10% (v/v) trichloroacetic acid and 40% (v/v) acetone followed by an incubation on ice for 10 min. The samples were centrifuged at 13,000 rpm for 5 min, spectra of the supernatants were recorded.

### 2.10 Steady-state kinetics

Apparent kinetic constants for the electron donors D-glucose, D-galactose and lactose were measured using the standard assay with ferrocenium. Kinetic constants for the electron acceptors DCIP, 1,4-benzoquinone and ferrocenium hexafluorophosphate were determined using 25 mM D-glucose as electron donor. The observed data were fitted to the Michaelis-Menten equation and kinetic constants were calculated by nonlinear least-squares regression (Sigma Plot 11, Systat Software, Chicago, IL). Turnover numbers (*k*_cat_) and catalytic efficiencies (*k*_cat_/*K*_m_) were calculated using the molecular mass.

### 2.11 O_2_ reactivity

The oxygen reactivity of the purified enzymes was determined using a fluorimetric assay with Amplex Red/horseradish peroxidase as described in section 2.6 with some modifications. The assay was carried out in black 96-well plates using 0.5 mg mL^−1^ of the purified enzymes, 50 µM Amplex Red, 25 mM D-glucose, 0.1 U mL^−1^ horseradish peroxidase and 50 mM sodium phosphate buffer pH 7.4. The increase in fluorescence of the wild-type, variant H103Y, two negative controls (assay without enzyme and assay without D-glucose) and an internal standard (2.5 µM hydrogen peroxide) was measured over 60 minutes and the slope per minute was determined. A calibration curve with hydrogen peroxide standards was prepared (Fig. S1) containing 50 µM Amplex Red, 50 mM sodium phosphate buffer pH 7.4 and hydrogen peroxide concentrations from 0–5 µM. Oxygen activity was calculated via the formed hydrogen peroxide per minute and milligram of enzyme by fitting the curve and solving the 3-parameter logarithmic equation.

## Results and Discussion

### 3.1 Expression of PDH in S. cerevisiae

The production of PDH from its native host organism *Agaricus sp*. is laborious and time-consuming [4], [5]. Therefore several attempts for heterologous expression of PDH in different organisms like *Aspergillus sp*. [37], *E. coli* and *P. pastoris* [36] were made. Due to glycosylation and solubility issues, PDH could only be sucessfully expressed in eukaryotic systems with *P. pastoris* being the host of choice for large-scale fast and efficient protein expression [36]. For protein engineering and mutagenesis, different requirements have to be considered. Expression of *pdh1* in *P. pastoris* using the constitutive *GAP* promoter yielded only low amounts of recombinant protein (our unpublished information), and using the strong inducible *AOX1* promoter requires repeated feeding with methanol, which is a cumbersome and contamination-prone procedure. Furthermore, vectors available for *P. pastoris* are integrated into the genome, yielding significantly fewer transformant colonies [38]. *S. cerevisiae* can maintain and replicate plasmids and is therefore the most commonly used eukaryotic organism for extensive mutagenesis studies [39]. In addition to higher transformation efficiencies, *S. cerevisiae* is able to perform *in-vivo* overlap-recombination of the vector and a library of inserts, which facilitates fast and simple library construction [40]. Therefore the expression of *A. meleagris pdh1* in *S. cerevisiae* under the control of the *GAL1* promoter in the vector pYES2/CT was established. The native signal sequence of PDH was used for expression in *S. cerevisiae,* as this was successful in other eukaryotic host organisms as well. With a combination of SC-dropout minimal medium as preculture medium to grow the cells to a uniform density and the SG/R-CAA expression medium containing the inducer D-galactose, activity levels high enough to sufficiently distinguish between active and inactive variants could be reached.

### 3.2 Site-saturation mutagenesis library construction and high-throughput screening

**Figure 1:**
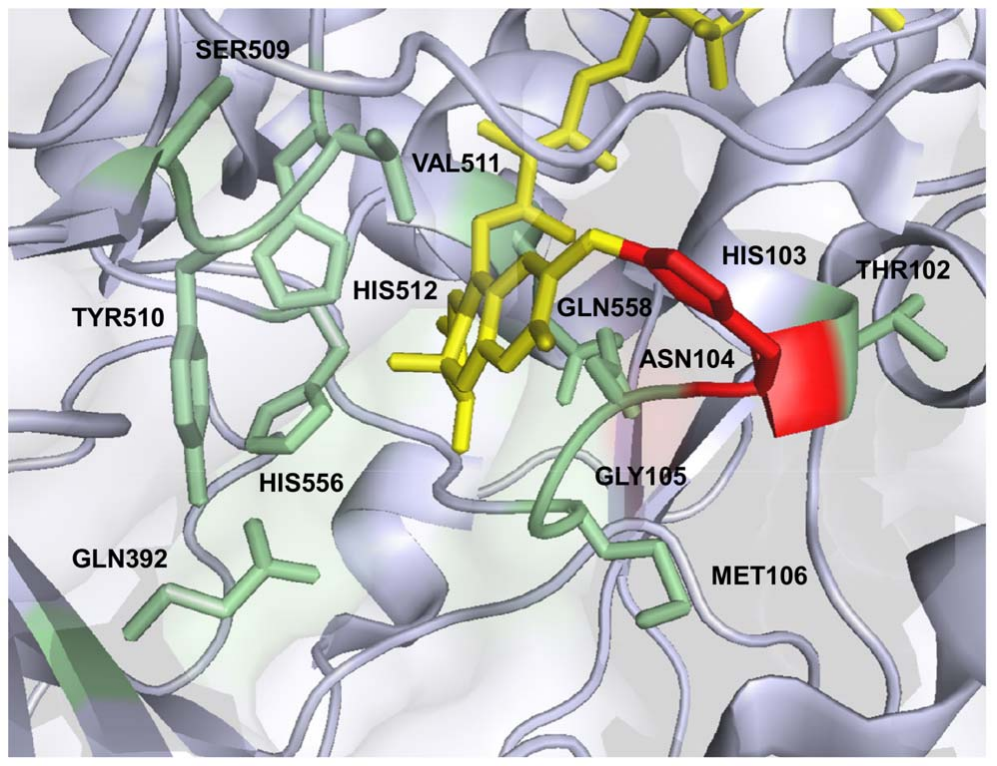
PDH active site. The covalently bound FAD cofactor is shown in yellow, the mutated amino acid positions (green) and HIS103 (red) are highlighted. The image was created using PyMOL (PDB code 4H7U), for clarity a few amino acids were omitted.

**Figure 2:**
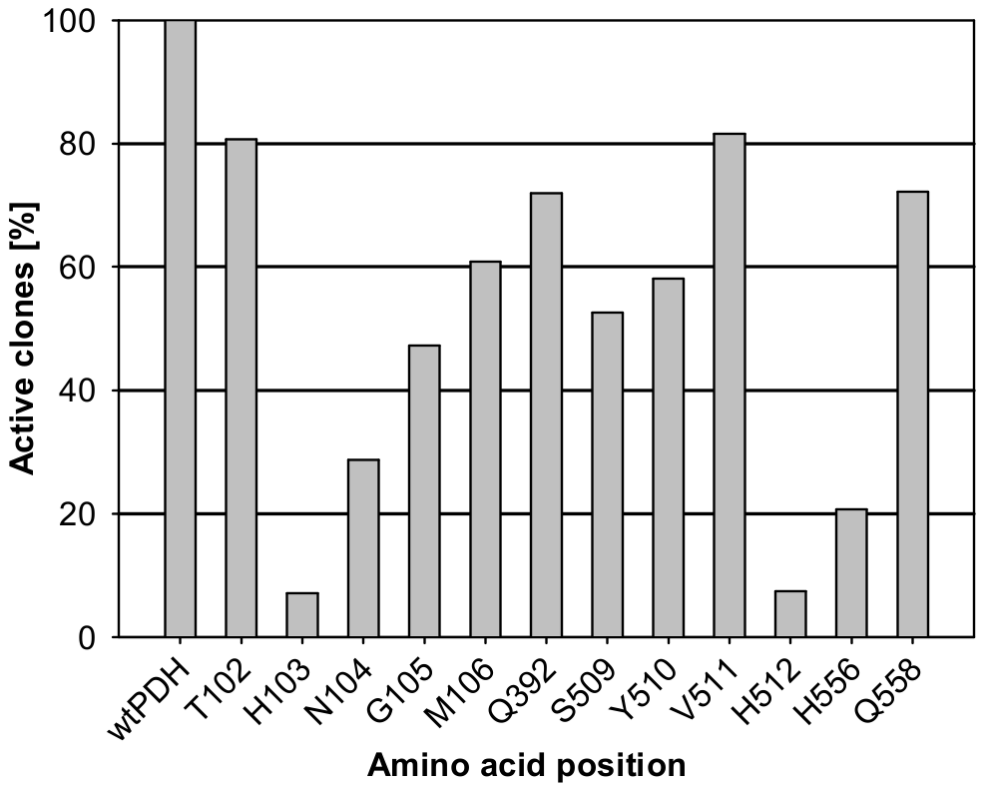
Active clones (in %) of site-saturation mutagenesis target positions. Activity was determined using a screening assay containing 0.3-glucose in 100 mM sodium acetate buffer pH 4 at 30°C. Libraries were expressed in *S. cerevisiae*. wt: wild-type *A. meleagris* PDH.

According to the crystal structure of *A. meleagris* PDH (PDB code 4H7U) twelve amino acids immediately surrounding the flavin isoalloxazine C(4a), the site of oxygen activation in flavoenzymes [14], [19], [41], and interacting with carbohydrate substrates in docking experiments [13] were targeted for site-saturation mutagenesis. Positions T102, H103, N104, G105, M106, Q392, S509, Y510, V511, H512, H556 and Q558 were selected (Fig. 1), and 352 clones were screened per position. Standard PDH activity was monitored with a DCIP-based assay and the libraries were screened for increased oxygen reactivity with an Amplex Red/horseradish peroxidase-based assay via the formed hydrogen peroxide. While the two catalytic histidines H512 and H556 are supposed to play an important role in the interaction of PDH with the sugar substrate and act as catalytic bases, H103 is the amino acid where the FAD cofactor is covalently bound to [13], [42]. Substitution of these three histidines resulted in the highest number of inactive variants in the screening using the DCIP/D-glucose assay with only 7.4% (H512), 20.7% (H556) and 7.1% (H103) active clones (Fig. 2), suggesting essential functions for these amino acids. Due to appropriate distances and hydrogen bonding interactions with the sugar both H512 and H556 showed the ability to act as catalytic base in molecular dynamics simulations of the interaction of AmPDH with D-glucose [42]. Graf and coworkers concluded that the two histidines could have similar roles to H502 and H546 in aryl-alcohol oxidase (AAO) [43], the GMC family member that shows the highest structural similarity to PDH, where H502 (H512 in PDH) is of higher importance for catalysis than H546 (H556 in PDH). This is reflected in the number of active variants with 7.4% for H512, which may all be represented by the wild-type amino acid, and 20.7% for H556. In *Trametes multicolor* POx, the equivalent of H556 is N593, indicating that asparagine can fulfill the same or similar functions at this position [44]. It is noteworthy that for H556 no variant with increased oxygen reactivity could be identified. In contrast, a mutation of the corresponding position N700 in *Myriococcum thermophilum* CDH to serine led to higher hydrogen peroxide production [33]. This illustrates that the generation of a favorable micro-environment for oxygen reactivity around the flavin C(4a)N5 locus is complex and cannot readily be achieved with single amino acid exchanges. All other amino acid positions showed a number of active variants higher than 50% except for N104 (28.7%) and G105 (47.3%). The highest variety of amino acids was tolerated at position T102 and V511 with more than 80% active clones. For position T102 this finding is not surprising as the side chain is pointing away from the FAD isoalloxazine but V511 occupies a prominent position in the active site, next to the catalytic histidine H512 (Fig. 1). Molecular dynamic simulation detected hydrogen bonding interaction of the sugar substrate with V511 but only with the backbone and not with the side chain [42]. This could be an explanation for the high tolerance for substitution of this very central amino acid position.

Variants with at least the two-fold oxygen reactivity of the wild-type were chosen for the rescreening. When the increased oxygen reactivity was confirmed in the rescreening, the amino acid exchange was identified by sequencing. All sequenced variants showed a substitution of the amino acid H103 to tyrosine, phenylalanine, tryptophan and methionine and an increase in oxygen reactivity of 2.0–2.6-fold in the screening compared to the wild-type AmPDH. These four amino acids have in common that they are all large to very large, aromatic (except for methionine), uncharged and (rather) hydrophobic [45]. All substitutions were encoded by all possible codons, a confirmation for the reliability of the mutagenesis technique. Due to the fact that the substitution to tyrosine was the most abundant and showed the highest increase in oxygen reactivity, variant H103Y was chosen for further characterization.

### 3.3 Large scale protein production and purification of PDH variant H103Y

**Figure 3:**
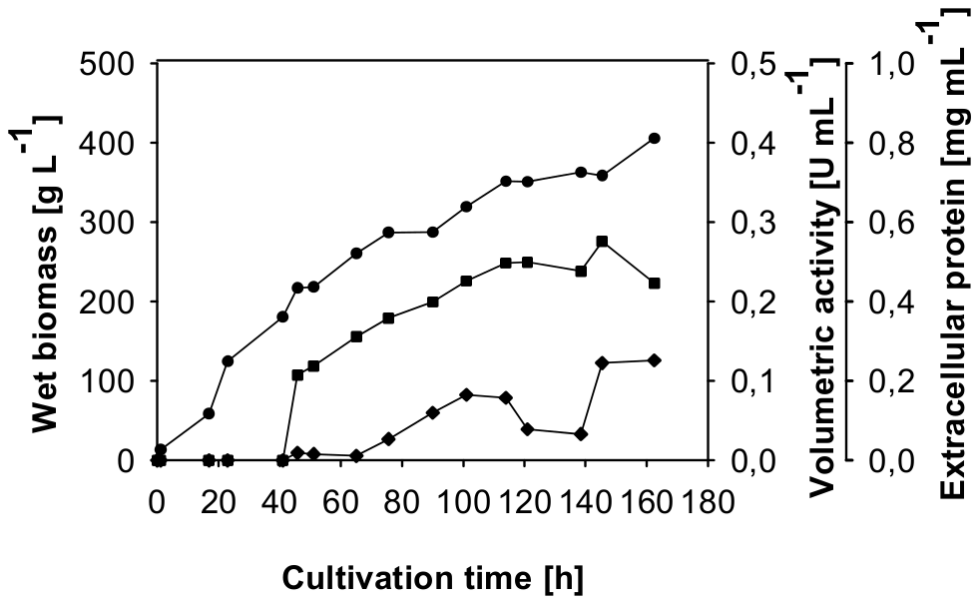
Large scale production of PDH variant H103Y in *P. pastoris*. Fermentation was carried out in a 7; squares, extracellular protein; diamonds, volumetric activity.

*P. pastoris* was chosen for the larger scale production of variant H103Y [36]. Variant H103Y was produced by site-directed mutagenesis and plasmid pPICZB_H103Y was transformed to competent *P. pastoris* X33 cells. A colony-PCR-verified clone of variant H103Y was used for large-scale protein production in a 7-L aerated and stirred bioreactor. During cultivation wet biomass, extracellular protein concentration and volumetric activity were monitored by taking samples in regular time intervals (Fig. 3). A level of wet biomass of 217 g L^−1^ could be obtained after the glycerol batch and fed-batch phase. The fermentation lasted for 163 h in total and a wet biomass of 405 g L^−1^, 0.45 mg mL^−1^ extracellular protein concentration and 0.13 U mL^−1^ of volumetric PDH activity were reached at time of harvest. The recombinant protein was purified in a 3-step protocol to apparent homogeneity, a hydrophobic interaction chromatography step was followed by anion exchange chromatography and a final gel filtration step (Table 1). The purified protein showed a bright yellow color, gel filtration pool 1 represents the purest enzyme preparation with a yield of 34% which was used for all further analyses. Together with the second pool 70% of the enzyme activity could be recovered. Recombinant wild-type AmPDH was produced in 60 L scale and purified as described in [36].

**Table 1:** Purification of PDH variant H103Y.

Purification step	Total protein	Total activity	Specific activity	Purification	Yield
	[mg]	[U]	[U mg^−1^]	[-fold]	[%]
Crude extract	1440.5	1746.2	1.21	1.00	100
Phenyl sepharose	407.4	1582.2	3.88	3.21	91
DEAE sepharose	209.1	1268.0	6.06	5.01	73
**Gel filtration pool 1**	**31.3**	**597.6**	**19.09**	**15.78**	**34**
Gel filtration pool 2	49.4	635.7	12.88	10.64	36

### 3.4 Molecular properties

**Figure 4:**
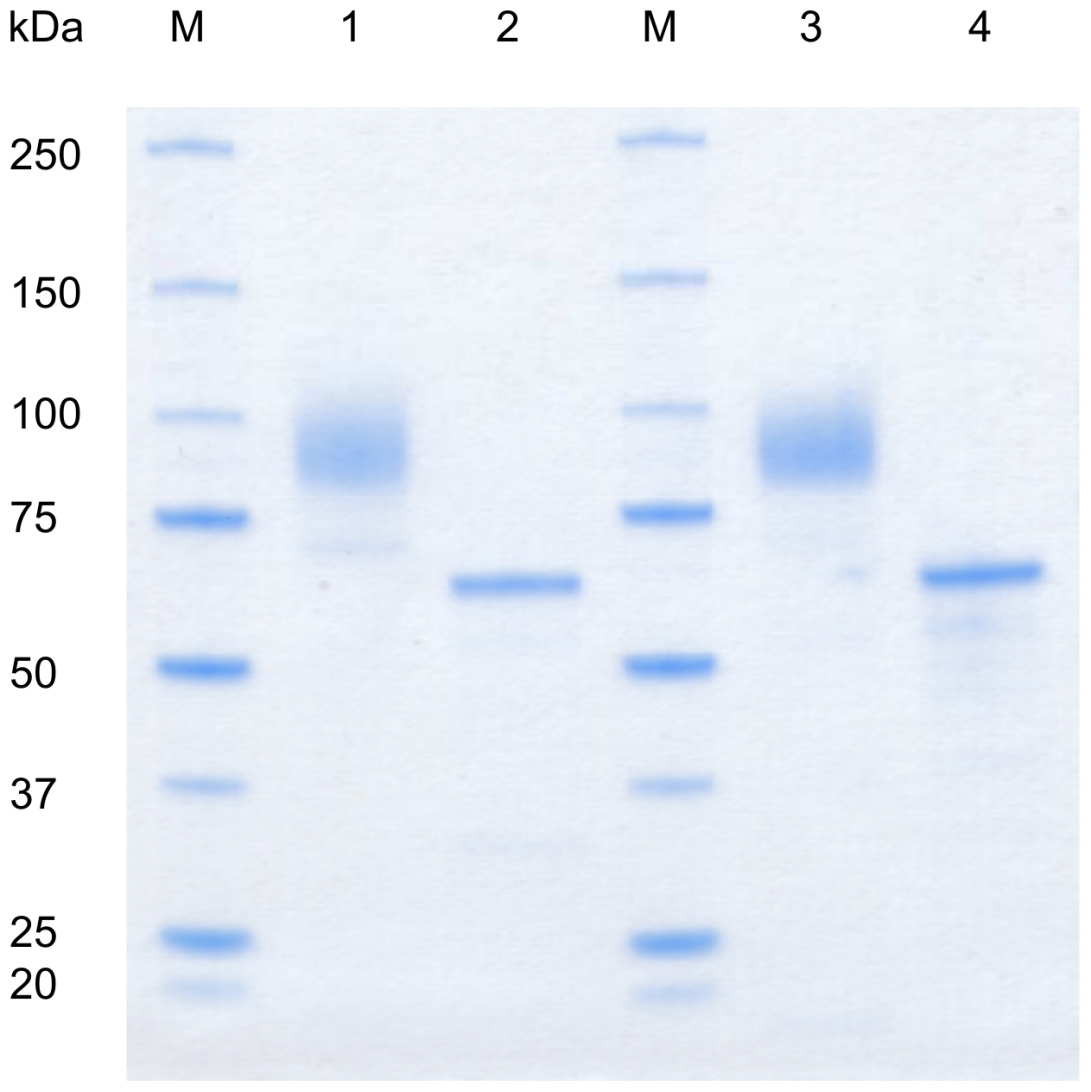
SDS-PAGE. Recombinant *A. meleagris* PDH and variant H103Y were expressed in *P. pastoris* and purified in a two- and three-step protocol. M, molecular marker (Precision Plus Protein Standard, BioRad); 1, wild-type *Am*PDH; 2, wild-type *Am*PDH deglycosylated (PNGase F); 3, *Am*PDH variant H103Y; 4, *Am*PDH variant H103Y deglycosylated.

Both the recombinant wild-type AmPDH and variant H103Y showed a broad smear around 90 kDa on SDS-PAGE (Fig. 4). Deglycosylation with PNGase F under denaturing conditions reduced the mass of both enzymes to around 64 kDa, which gives a degree of glycosylation of around 30%. This value is in good agreement with other PDH glycoproteins produced so far in *P. pastoris* [6], [36]. The high degree of glycosylation is due to the additon of glycans of the high-mannose type by the yeast [46]. The molecular properties of the mutant appear unchanged in SDS-PAGE compared to the wild-type.

**Figure 5:**
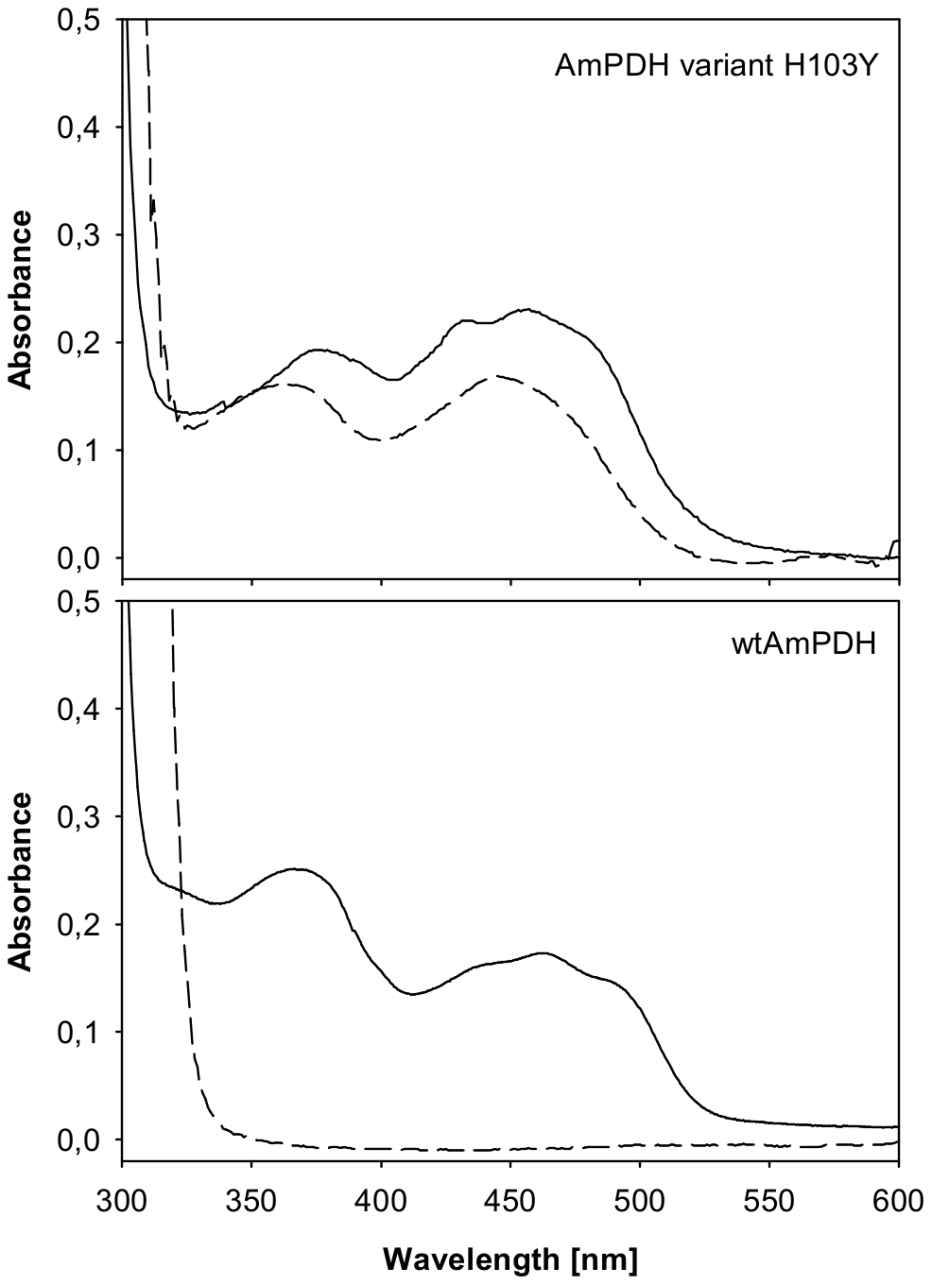
TCA/acetone precipitation of wild-type AmPDH and variant H103Y. Precipitation was carried out using 10% TCA and 40% acetone on ice, spectra of the oxidized proteins before (solid lines) and the supernatants after precipitation are shown (dashed lines).

The mutation of H103 to a tyrosine raised the question whether the FAD-cofactor was still covalently attached to the enzyme. The proteins were precipitated with 10% TCA and 40% acetone and spectra of the protein solution before and the supernatant after the treatment were recorded (Fig. 5). Due to the colorless supernatant lacking the flavin peak and the bright yellow protein pellet it could be clearly seen that the wild-type protein did not release the covalently bound FAD. Precipitation of variant H103Y resulted in a bright yellow supernatant with a spectrum typical for free FAD and a colorless protein precipitate. In none of the purification steps described above yellow fractions without PDH activity could be found, confirming that the mutant did not lose FAD during purification. When stored at 4°C, the activity of the mutant remained stable over several weeks, otherwise this would be a sign for weak incorporation of the FAD cofactor. These findings indicate that the variant H103Y carries a tightly but non-covalently bound FAD cofactor.

Comparing the spectra of the unprecipitated proteins, a shift towards blue (hypsochromic shift) of the second flavin peak (around 370 nm) can be observed for the wild-type enzyme. This is typical for an 8α-modified FAD in contrast to the unmodified cofactor of variant H103Y [47]. A covalent bond formation in PDH specific for (the N3 of) H103 can be concluded, which is not possible with a tyrosine at this position. A similar observation was made for vanillyl-alcohol oxidase (VAO) [48].

### 3.5 Steady-state kinetics

To examine the effect of the mutation, steady-state kinetics for electron donors were determined using ferricenium as the electron acceptor (Table 2). Catalytic efficiencies (*k*_cat_/*K*_m_) for the monosaccharides D-glucose and D-galactose are around eight-fold lower for variant H103Y compared to the wild-type due to higher *K*_m_-values and lower turnover numbers (*k*_cat_). The mutation also decreased the catalytic efficiency for the disaccharide lactose about three-fold, indicating a negative effect of the mutation on sugar substrate turnover. A disruption of the covalent FAD-linkage leads in most cases to a decrease in reduction potential of the mutant and therefore impaired substrate-mediated flavin reduction [48]–[50]. AmPDH has a relatively high midpoint potential of +150 mV [51], it is very likely that non-covalent mutant H103Y exhibits a lower potential. A detailed electrochemical characterization of the mutant H103Y is a matter of ongoing studies.

**Table 2:** Apparent kinetic constants of recombinant *A. meleagris* PDH and variant H103Y for selected electron donors, determined with 0.2 mM ferricenium as electron acceptor at 30°C.

	AmPDH^a^			H103Y		
	*K*_m_	*k*_cat_	*k*_cat_/*K*_m_	*K*_m_	*k*_cat_	*k*_cat_/*K*_m_
	[mM]	[s^−1^]	[mM^−1^ s^−1^]	[mM]	[s^−1^]	[mM^−1^ s^−1^]
D-glucose	0.69±0.09	37.8±1.1	54.8	3.85±0.35	27.4±0.5	7.1
D-galactose	1.07±0.13	47.3±3.0	44.2	5.29±0.39	28.9±2.1	5.5
Lactose	128±11.9	41.0±8.1	0.32	395.90±16.27	24.7±0.4	0.1

a Data from [36].

Kinetic constants for electron acceptors were measured with 25 mM D-glucose as the electron donor (Table 3). Catalytic efficiencies of variant H103Y for electron acceptors other than oxygen are generally lower compared to the wild-type protein. For ferrocenium hexafluorophosphate and DCIP variant H103Y showed a lower *K*_m_-value (higher affinity) but also a lower turnover number (*k*_cat_) than the wild-type AmPDH. Catalytic efficiencies are reduced around three- and two-fold for these electron acceptors whereas 1,4-benzoquinone showed a nearly twelve-times reduced catalytic efficiency, due to a higher *K*_m_-value of variant H103Y for 1,4-benzoquinone.

**Table 3:** Apparent kinetic constants for selected electron acceptors and oxygen reactivity of recombinant *A. meleagris* PDH and variant H103Y, determined with 25 mM D-glucose as electron donor at 30°C.

	AmPDH			H103Y		
	*K*_m_	*k*_cat_	*k*_cat_/*K*_m_	*K*_m_	*k*_cat_	*k*_cat_/*K*_m_
	[mM]	[s^−1^]	[mM^−1^ s^−1^]	[mM]	[s^−1^]	[mM^−1^ s^−1^]
Fc^+^PF_6_ (pH 8.5)	0.16±0.04^a^	130±11^a^	812.5	0.10±0.01	26.0±0.9	260.0
1,4-BQ (pH 4)	1.38±0.28^a^	65.4±5.5^a^	47.4	3.64±0.47	13.6±1.1	4.0
DCIP (pH 4)	0.14±0.01	40.7±4.1	290.7	0.02±0.01	2.8±0.5	140.0
O_2_ [µM min^−1^ mg^−1^]	0.095±0.003			0.500±0.033		

a Data from [36].

**Figure 6:**
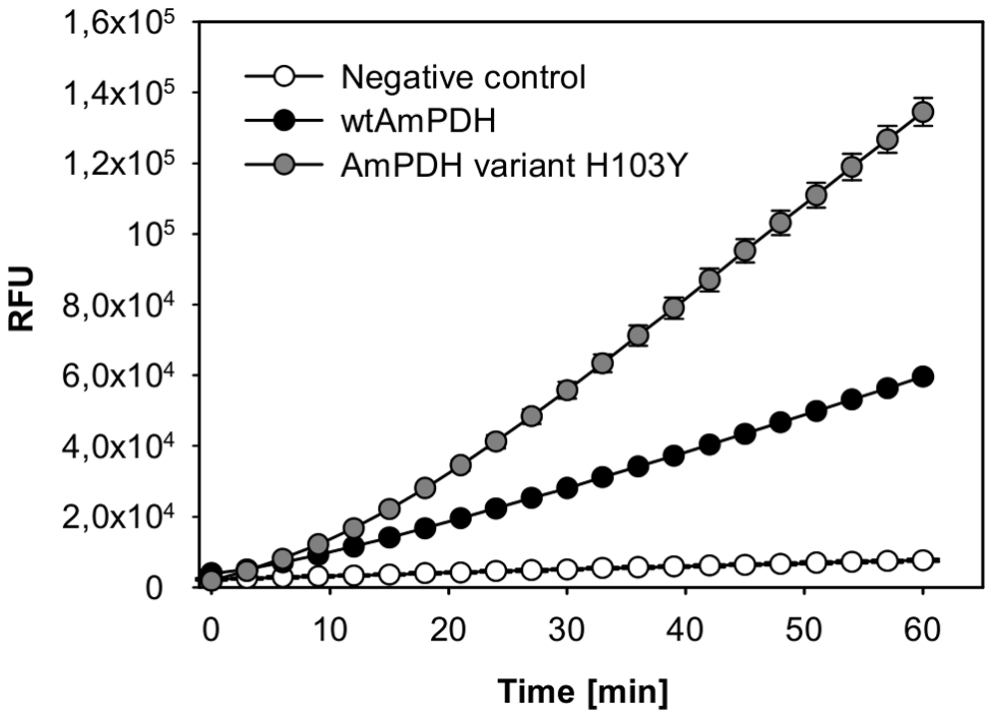
Oxygen reactivity of wild-type AmPDH and variant H103Y. Oxygen reactivity was determined using an Amplex Red/horseradish peroxidase based assay. The assay contained 0.5 mg/mL of the purified enzyme, 50 µM Amplex Red, 25 mM D-glucose, 0.1 U mL^−1^ horseradish peroxidase and 50 mM sodium phosphate buffer pH 7.4. Error bars represent the standard deviation of three repeats.

Oxygen reactivity was assessed indirectly via the formed hydrogen peroxide in the Amplex Red/horseradish peroxidase fluorimetric assay. To calculate the amount of H_2_O_2_, a calibration curve was established and a 3-parameter logarithmic function (y = y0+a*ln(x−x0)) turned out to give the best fit (R^2^ = 0.999). The standard curve showed a slight offset due to background fluorescence (Fig. S1). Wild-type AmPDH produced 0.095 µM of hydrogen peroxide per mg enzyme and minute whereas variant H103Y produced 0.500 µM per mg and minute, representing a 5.3-fold increase in oxygen reactivity (Table 3, Fig. 6). As the fluorimetric assay of the protein samples did not start with identical RFU values (depending on sample handling times and “dead time” of the plate reader), the slope per minute was used for the calculation of the formed hydrogen peroxide. The calibration curve is not linear and flattens off towards higher hydrogen peroxide concentrations, therefore the more than five-fold increase in oxygen reactivity cannot be directly estimated from Figure 6. Due to the still low reactivity of PDH with oxygen, catalytic constants (*K*_m_, *k*_cat_) could not be determined.

Position H103 is located on the *si*-side of the flavin isoalloxazine and covalently links the cofactor to the enzyme. A disruption of this covalent bond could lead to an altered arrangement of the FAD isoalloxazine in the active site and create more space or form channels to facilitate O_2_ diffusion. In a study of Hernández-Ortega and coworkers [52], the replacement of a phenylalanine located in a narrow oxygen channel of AAO by alanine decreased oxygen reactivity substantially. A substitution with an even bulkier tryptophan resulted in a two-fold increase, suggesting that volume restrictions as determinants of oxygen reactivity should be viewed with care. Only mostly unpolar, uncharged residues (tyrosine, phenylalanine, tryptophan and methionine) were found replacing H103 in oxygen-active clones. This points towards the importance of a nonpolar site near the flavin C(4a), as observed for choline oxidase [22]. As already mentioned in the introduction, in the recently published crystal structure of AmPDH, a C(4a)-adduct on the *si*-side of the isoalloxazine was observed. This observation shows that AmPDH is able to stabilize this oxygen species which is tightly coordinated by the catalytic histidines H512 (Nε2) and H556 (Nδ1). The distance between this catalytic pair is shorter in AmPDH (3.5 Å) than in TmPOx (4.2 Å), which was found to show a physiological C(4a) adduct [13], [16]. The shorter distance might lead to an “overstabilization” of the C(4a)-hydroperoxide and limit oxygen reactivity in wild-type AmPDH, the non-covalently bound FAD in AmPDH H103Y variant could create more space and lead to a destabilizing effect, therefore increase oxygen reactivity. Crystallographic data of mutant H103Y would be of interest to support this hypothesis. In summary, the study provides a starting point for engineering efforts with PDH and valuable insight on the catalytic importance of the amino acid environment surrounding the FAD cofactor of PDH. The large distance of position H103 to the C(4a) and the lack of a crystal structure of the H103Y variant make providing a rationale for the increased oxygen reactivity challenging. Further engineering attempts and studies towards increased or altered oxygen reactivity of PDH and other flavoproteins will be required.

## Supporting Information

Figure S1 **Calibration curve H_2_O_2_ Amplex Red assay.** The curve was fitted with a 3-parameter logarithmic function (y = y0+a*ln(x-x0)). The assay contained the indicated concentration of hydrogen peroxide, 50 µM Amplex Red, 0.1 U mL^−1^ horseradish peroxidase and 50 mM sodium phosphate buffer pH 7.4. Error bars show the standard deviation of six repeats.
(TIF)

Table S1 **Nucleotide sequences of the primers.** Sites of restriction or mutagenesis are indicated in bold letters. N = A, T, G, C; K = G, T.
(DOCX)
