# Supplementary material for: Engineering of Pyranose Dehydrogenase for Increased Oxygen Reactivity
Source: PLoS One. 2014 Mar 10;9(3):e91145. doi: 10.1371/journal.pone.0091145 (PMC3948749; doi:10.1371/journal.pone.0091145)
Supplement: Table S1 — Nucleotide sequences of the primers. Sites of restriction or mutagenesis are indicated in bold letters. N = A, T, G, C; K = G, T. (DOCX) [file pone.0091145.s002.docx]

**Table S1: Nucleotide sequences of the primers.** Sites of restriction or mutagenesis are indicated in bold letters. N=A, T, G, C; K=G, T.

| Name | Sequence 5’ – 3’ |
| --- | --- |
| AmKpnIfw | CATAG**GGTACC**ATGCTGCCTCGAGTGACCA |
| AmBamHIfw | ATTAA**GGATCC**ATGCTGCCTCGAGTGACCA |
| AmXbarev | CGTGC**TCTAGA**TTAGTTATAACTCTTTG |
| AmNotIrev | CGTGC**GCGGCCGC**TTAGTTATAACTCTTTG |
| AmFw | CCTTCGAGTGGCAAGAATTCACCACATATT |
| AmRev | CACTCGAAGG**GTC**CTGTCCGTTCAGTTTG |
| AmT102fw | cctgggtggctgcagt**nnk**cataatggaatggtg |
| AmT102rev | caccattccattatg**mnn**actgcagccacccagg |
| AmH103fw | gtggctgcagtact**nnk**aatggaatggtgtac |
| AmH103rev | gtacaccattccatt**mnn**agtactgcagccac |
| AmN104f | gctgcagtactcat**nnk**ggaatggtgtacacccg |
| AmN104rev | cgggtgtacaccattcc**mnn**atgagtactgcagc |
| AmG105fw | gcagtactcataat**nnk**atggtgtacacccgag |
| AmG105rev | ctcgggtgtacaccat**mnn**attatgagtactgc |
| AmM106fw | gtactcataatgga**nnk**gtgtacacccgaggtt |
| AmM106rev | aacctcgggtgtacac**mnn**tccattatgagtac |
| AmQ392fw | ccacatattgagttc**nnk**tttgcacaaatcacc |
| AmQ392rev | ggtgatttgtgcaaa**mnn**gaactcaatatgtgg |
| AmS509fw | gcgatcttcaacattc**nnk**tacgtgcatggtgt |
| AmS509ev | acaccatgcacgta**mnn**gaatgttgaagatcgc |
| AmY510fw | cttcaacattctca**nnk**gtgcatggtgtggg |
| AmY510rev | cccacaccatgcac**mnn**tgagaatgttgaag |
| AmV511fw | caacattctcatac**nnk**catggtgtgggaacg |
| AmV511rev | cgttcccacaccatg**mnn**gtatgagaatgttg |
| AmH512fw | cattctcatacgtg**nnk**ggtgtgggaacgttg |
| AmH512rev | caacgttcccacacc**mnn**cacgtatgagaatg |
| AmH556fw | catgctccggccgca**nnk**actcaactacctgt |
| AmH556rev | acaggtagttgagt**mnn**tgcggccggagcatg |
| AmQ558fw | ggccgcacacact**nnk**ctacctgtttacgca |
| AmQ558rev | tgcgtaaacaggtag**mnn**agtgtgtgcggcc |
| H103Yfw | aatggaatggtgtacacccgaggttc |
| H103Yrev | ccattccatt**ata**agtactgcagccaccca |
